# Too hard, too easy, or just right? The effects of context on effort and boredom aversion

**DOI:** 10.3758/s13423-024-02528-x

**Published:** 2024-06-05

**Authors:** Jake R. Embrey, Alice Mason, Ben R. Newell

**Affiliations:** 1 School of Psychology, UNSW, Sydney, Australia; 2 Department of Psychology, University of Bath, Bath, UK; 3 Institute for Climate Risk & Response, UNSW Sydney, Sydney, Australia

**Keywords:** Cognitive effort, Boredom, Opportunity costs, Decision-making

## Abstract

Despite people’s general desire to avoid cognitive effort, there is a limit to our parsimony: boredom, a state defined by a lack of successful mental engagement, is found to be similarly aversive. The work presented here investigates how context – the alternative tasks present and the environmental context – impacts people’s aversion to exerting cognitive effort and avoiding boredom via a demand-selection task. In a population of undergraduate students, we assessed how people’s willingness to exert mental effort (in a working memory task) is affected by the presence of an easier alternative (less cognitively demanding) or a boring alternative (doing nothing at all). To manipulate environmental context, we conducted the experiment online, where participants completed the task remotely, and in a controlled laboratory setting. We find people willingly seek out effortful tasks to avoid boredom, despite avoiding high demands when both tasks on offer required some effort. We also find large effects of the participants’ environmental context, with preferences for the most demanding task increasing by over 150% in the lab compared to online. These results bear relevance to theories that argue the costs of effort are determined relative to the alternatives available (e.g., opportunity cost theories). Moreover, the results demonstrate that researchers who deliberately (or inadvertently) manipulate effort and boredom must consider the effects context (both choice and environmental) may have on people’s behaviour.

**Supplementary information:**

The online version contains supplementary material available at 10.3758/s13423-024-02528-x.

## Introduction

When money, time, and all else is equal, people are typically averse to exerting mental effort. In scenarios that demand our cognitive faculties we tend to abide by Hull’s (1943) ‘law of less work’, opting for the least effortful means to our desired end. In some instances, this desire to avoid effort is strong enough that we are willing to forgo rewards – such as the aforementioned money and time (e.g., Embrey et al., 2023; Kool et al., 2010; Oprea, 2020) – to minimise the effort we exert. As Fiske and Taylor (1991) put it, we are ‘cognitive misers’… but perhaps not all of the time.

Boredom, which occurs when people are unable to effectively engage their cognitive faculties (Eastwood et al., 2012; Elpidorou, 2018), is often found to be as aversive as situations that impose high cognitive demands. To avoid states of boredom, people unsurprisingly seek enjoyable alternatives such as listening to a podcast on their commute. People, however, also seek negative experiences such as receiving electric shocks (Bench & Lench, 2019) or viewing disgusting images (Nederkoorn et al., 2016) to relieve boredom.

In this paper we examine the trade-off between effort and boredom aversion. The central idea we explore is that the relative aversion to boredom and effort is a function of the alternative activities available in the environment (Struk et al., 2020). A maths task might be aversive when the alternative is watching TV, but if the only alternative is staring at a blank wall, then maybe the maths is not so bad.

The idea that people’s aversion to boredom and effort are a function of available alternatives highlights both the limits of the cognitive miser account (Fiske & Taylor, 1991) and the importance of context on people’s willingness to exert mental effort (for other examples of context effects on effort, see Ashburner & Risko, 2022; Desjardins et al., 2023; Otto & Vassena, 2021; Otto et al., 2022). According to opportunity cost theories of effort aversion (Kurzban et al., 2013; Kurzban, 2016), the reason mental exertion is experienced as costly is because the ‘sense of effort’ experienced indexes the value of the current task relative to available alternatives, rather than simply indexing the raw computational resources demanded by a task. Relatedly, Agrawal and colleagues (2022) propose an opportunity cost theory of both effort and boredom aversion; they argue our sense of effort is a signal that the value of rest outweighs the benefits of continued task engagement, whereas boredom is a signal that indicates more fruitful alternatives may be available.

In the current experiments we examine such opportunity cost accounts by assessing how individuals’ preferences for effortful and boring options change as the available alternative tasks become either more or less cognitively demanding, and the context in which they are offered affords more or less opportunity to do something else. We do this by offering participants choices between doing easier or harder working-memory tasks – or doing nothing – both in the lab and online.

Our work builds directly on recent research by Wu and colleagues (2023) and extends it in two key ways. Across separate experiments, Wu and colleagues found when given the choice between an easy or a difficult working memory task, people preferred the easy task; when given the choice between ‘doing nothing’ and the easy task, people were indifferent; but when choosing between ‘doing nothing’ and the difficult task, people preferred the hard task despite ‘doing nothing’ being the truly *effortless* alternative. One interpretation of these results is that boredom and effort have a similar influence on choice and that people perceive boredom to be as costly as exerting effort.

A limitation of this work, however, is that participants in Wu et al.’s experiments were only ever offered the choice between two alternatives. This design restricts the conclusions that can be drawn about the *relative* attractiveness of different levels of effort and boredom as a function of the tasks on offer. Here we use a within-subjects design where individual participants made choices between all possible task comparisons – easy or hard, do nothing or easy, do nothing or hard. This design, in contrast with the between-subjects experiments of Wu et al. (2023), allows insight into how individual preferences shift across different choice contexts; for instance, how an individual’s preference for the harder task changes when the alternative is the effortless ‘do nothing’, as opposed to the easier task.

The second key extension of our approach is to conduct a direct comparison of the preference for effort over boredom in contexts that differ in terms of the availability of other options: online versus in the lab. This comparison is critical if we want to assess the real opportunity cost involved in a choice to ‘do nothing’.^1^ When experiments entail the manipulation of cognitive demand or boredom, a participant’s broader environment (i.e., things outside the confines of the experiment) is likely to affect their preferences. Inducing boredom by asking a participant to ‘do nothing’ in a lab is markedly different to doing so when the participant is in the comfort of their own home. Attentionally demanding tasks such as those used in mental effort research (e.g., working memory tasks) are also susceptible to distractions (for mobile phones as distractors, see Ito & Kawahara, 2017; Ward et al., 2017; for general reviews, see Oberauer et al., 2018; Lorenc et al., 2021). Distractions such as phones, people, and other extraneous influences can be controlled in the lab, but are unmonitorable when participants complete a task online.

Our experiments present participants, all drawn from the same university sample, with choices between tasks that require no effort, low effort, or modest effort to complete. We conducted the same experiment online and in the lab, in the same timeframe, thus varying only the location in which tasks were completed. This combination of design features allows us to shed more light on exactly when and how the costs of boredom and effort vary with the value of available alternatives both within and outside the experimental context.

Following the findings of Wu et al. (2023), we expected to find that participants’ preference for a harder working memory task would be stronger when the alternative was the effortless doing nothing, as opposed to the less-demanding working memory task – that is, people’s aversion to effort would be counteracted by their aversion to boredom. We also expected to see an overall increase in the preference for effortful tasks when the opportunity of being able to do something else is reduced by virtue of conducting the study in the lab.

While choice preferences changing as a function of the alternatives broadly aligns with opportunity cost models of effort, such models also predict that the phenomenology of exertion during a task and subsequent on-task behaviour will be affected by the value of the alternatives. To examine this prediction, we assessed participants’ subjective ratings of boredom and effort for each task throughout the experiment, as well as their accuracy to determine whether on-task behaviour changed alongside choice preferences.

## Method

All experimental data and analysis scripts can be accessed via the Open Science Framework at https://osf.io/e86fq/.

### Participants

We aimed to obtain sample sizes comparable to those of Wu et al. (2023), who observed medium effect sizes (Cohen’s *d* ~ 0.50) – keeping in mind our design was within-subjects.

Seventy participants signed up for and completed the Online Experiment via the University of New South Wales’ (UNSW’s) SONA system (*M*_age_ = 19.76 years, *SD*_age_ = 2.61 years; 38 females, 25 males, seven preferred not to answer). This experiment was conducted in March-April 2022.

Fifty-six participants signed up for and completed the Lab Experiment at UNSW’s Psychology department (*M*_age_ = 18.93 years, *SD*_age_ = 1.58 years; 29 females, 20 males, five preferred not to answer, two non-binary). This experiment was conducted in June 2022.

All participants were enrolled in first year psychology courses at UNSW and were compensated with course credit. Participants did not receive additional compensation or incentivisation for their decisions in the task. The experiment was approved by the UNSW School of Psychology Ethics Committee (Approval number: HREAP 3477).

### Materials

#### Online experiment

Participants completed the experiment on their own laptop or desktop computer. Mobile devices were not permitted. The experiment was coded in jsPsych (de Leeuw, 2015) and JavaScript.

#### Lab experiment

Participants completed the experiment in UNSW’s Cognition Lab on HP Desktop computers. Each participant completed the experiment alone in a testing room that contained only a computer. Participants’ mobile phones were given to the experimenter while they completed the task. The experiment was coded in jsPsych (de Leeuw, 2015) and JavaScript.

### Design

#### Do nothing and Add-N tasks

The online and lab-based experiment consisted of three types of tasks: the Do-Nothing task, plus the Add-1 and Add-3 tasks. Each of these different tasks was associated with a different coloured deck of cards (red, blue, or green deck).

The Do-Nothing task consisted of, as the name implies, doing nothing. Specifically, participants saw a prompt on the screen, “do nothing”, for 10 s and were required to sit and wait for the trial to end.

The Add-1 and Add-3 tasks were introduced by Kahneman et al. (1968) and later used by Wu and colleagues (2023). In both versions, four random numbers between 1 and 9 were displayed sequentially and participants were required to add 1 or add 3 to each number, depending on the task. For example, if the numbers **3 7 1 9** were displayed, the correct answer in the Add-1 would be **4 8 2 0**, and **6 0 4 2** in the Add-3 task. Each number was displayed for 850 ms with a 150-ms inter-trial interval (ITI). After all numbers were displayed sequentially, participants had 5 s to type their answer.^2^ Backspacing and re-typing answers was allowed within this 5-s period.

#### Demand selection task structure

The experiment consisted of six blocks, each of which contained ten repeated choices between the two tasks on offer. There were two blocks each of Do Nothing or Add-1, Do Nothing or Add-3, and Add-1 or Add-3, presented in a random order. There were therefore 20 choices for each task comparison and 60 choices total for each participant. The two options were always labelled so participants did not need to learn the deck-task association.

The number of trials per comparison (i.e., 20) is less than the number typically used in Wu et al. (2023). The within-subjects design also meant there was increased novelty from the participant’s perspective as the two offered options changed over blocks. This was not a feature of Wu et al. (2023).

#### Demand and boredom self-report ratings

After each block, participants were asked two self-report questions for each task they had seen in the previous block: “how mentally demanding was the [task type] deck?” and “how boring was the [task type] deck?” Ratings were obtained on a 7-point Likert scale, with 1 indicating low demand or boredom, and 7 indicating the highest level of demand or boredom. If participants chose only one type of task during a block, they were asked only about that task type and not the unchosen alternative.

### Procedure

Participants were provided with instructions describing how the Add-1, Add-3, and Do-Nothing tasks worked. To ensure participants understood all tasks they were given the opportunity to practice each task, receiving accuracy feedback for the two Add-N tasks.

Once participants completed the instructions and practice phase, they were told they would need to choose between the different tasks. Participants were explicitly instructed that if one type of deck (task) began to feel preferable, they could choose that deck more often. Furthermore, participants were informed that both tasks took the same amount of time and the experiment’s total time could not be shortened by choosing one task over another. Participants did not receive accuracy feedback in this phase.

Upon completion of the six choice blocks (two of each comparison with self-report ratings following each block), participants were asked “Did you do anything else, e.g., switch to another tab, look at your phone during this study?” and told they would receive their course credit regardless of response.

All instructions and debriefings were presented on the computer for both the Online and Lab Experiment to minimise potential confounds.

## Results

### Exclusion criteria

Prior to analysis we identified the number of participants who self-reported doing something else during the experiment. Thirteen participants self-reported in the Online experiment and one participant in the Lab experiment.^3^ We removed these participants from the analysis.

Following precedent (Wu et al., 2023) we also removed participants who had less than 25% average accuracy for either of the Add-N tasks. This led to the removal of a further eight participants from the Online experiment and four from the Lab Experiment. The samples sizes for the Online and Lab Experiments analysed below are 49 and 51, respectively.

Results retaining the full sample (i.e., no exclusion criteria) are reported in the Appendix. To summarise, the removal of these participants had no meaningful effect on the results nor our interpretation. Any minor changes are noted in the Results section.

### Accuracy

We calculated accuracy on a per trial basis with participants required to get all four numbers correct in their response on the Add-1 and Add-3 tasks. If one or more numbers were incorrect, their response for that trial was coded as incorrect (cf., Wu et al., 2023 for an identical procedure).

**Fig. 1:**
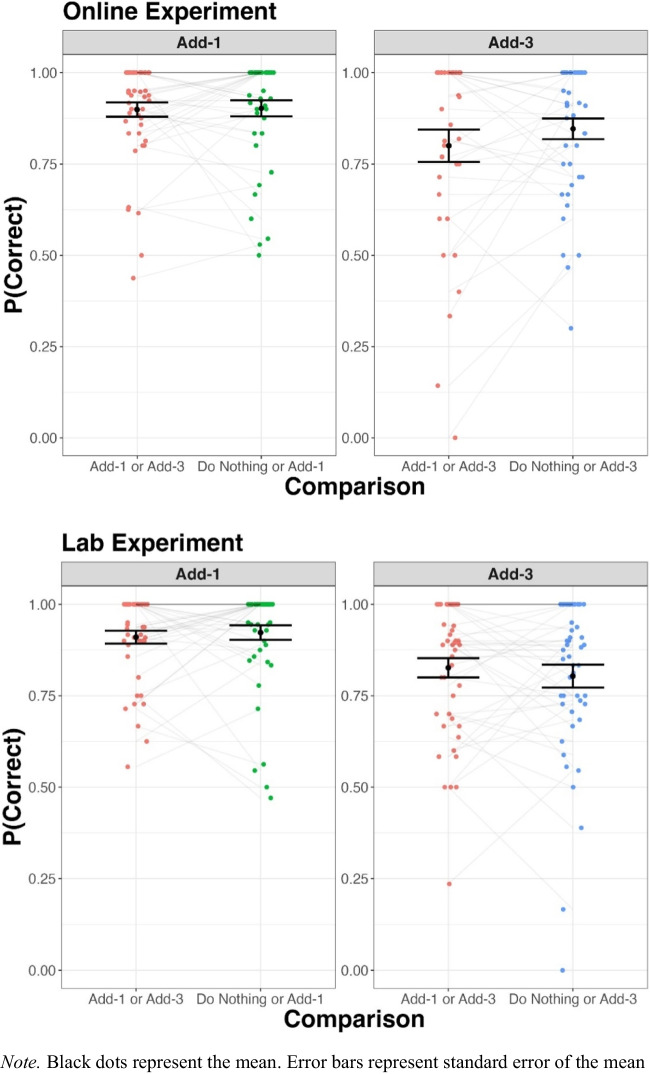
Average Add-1 and Add-3 accuracy, per comparison, for the Online and Lab Experiment

Average accuracy, split by comparison, is shown in Fig. 1. Across both experiments participants were generally better at the Add-1 than the Add-3 task. The effect of comparison on accuracy was assessed using a generalised linear mixed model (*lme4* package in R; Bates et al., 2015) separately for both the Online and Lab experiment. The models were fit to per trial data with accuracy as a binary variable (1 = correct, 0 = incorrect). The addition of *comparison* – *correct ~ comparison* + (*comparison* | *participant* ) – to an intercept only model did not improve the model fit for either the Add-1 or Add-3 asks in both the Online and Lab experiments. See the Appendix for full analysis details.

### Demand selection task preferences

#### Choice context effects

**Fig. 2:**
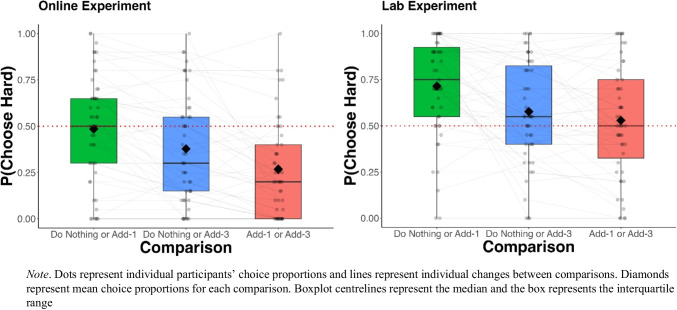
Average choice proportions for the three comparisons for both online and lab experiments

Average choice preferences for both the Online and Lab experiments are presented in Fig. 2.

To assess how preferences for the harder option changed as a function of task comparison (e.g., Do Nothing or Add-1), we fit a generalised linear mixed model *choice ~ comparison +* (1*| participant*) and compared it to a baseline, intercept only model. The *choice* response variable was a binary response: 0 (easy option) and 1 (hard option). The hard option was the Add-N tasks when the alternative was Do Nothing, and Add-3 when the alternative was Add-1. These models were fit separately for each experiment.

In both the Online and Lab experiments the addition of *comparison* significantly improved model fit, indicating people’s choices for the more demanding task were affected by the comparison type (Table 1; including Akaike weights, Wagenmakers & Farrell, 2004). Follow-up pairwise contrasts showed preferences for the harder option were significantly different for all three pairwise comparisons in both experiments (Table 2). These results indicate that Do Nothing was preferred more when the alternative was Add-3 as opposed to Add-1, but people preferred the Add-3 task more when the alternative was Do Nothing compared to when it was Add-1.

**Table 1:** Results for generalised linear mixed model (GLMM): online and lab experiments

Model	AIC	Akaike Weight
Online experiment		
*choice ~* 1* +* (1*| participant*)	3171.316	< .001
*choice ~ comparison +* (1*| participant*)	3039.373	> .999
Lab experiment		
*choice ~* 1* +* (1*| participant*)	3457.261	< .001
*choice ~ comparison +* (1*| participant*)	3355.597	> .999

**Table 2:** Results for pairwise contrasts: online and lab Experiments

Contrast	Estimate (SE)	z-score	*p*-value
Online experiment			
(A-1 or A-3) – (Nothing or A-1)	-1.310 (.115)	-11.354	< .0001
(A-1 or A-3) – (Nothing or A-3)	-.689 (.114)	-6.026	< .0001
(Nothing or A-1) – (Nothing or A-3)	.621 (.109)	5.685	< .0001
Lab experiment			
(A-1 or A-3) – (Nothing or A-1)	-1.055 (.108)	-9.752	< .0001
(A-1 or A-3) – (Nothing or A-3)	-.259 (.102)	-2.524	.0348
(Nothing or A-1) – (Nothing or A-3)	.796 (.108)	7.375	< .0001

Estimates are given on the log odds ratio, not the response variable scale. *p*-values are Bonferroni corrected

Individual preferences for the harder option were also correlated across comparisons for both the Online and Lab Experiments (see correlation matrices in the Appendix) suggesting people’s appetite for effort was consistent across comparisons.

Participants’ preferences were generally transitive and did not directly align with those observed by Wu et al. (2023): in our experiment, people’s preferences for the hard task (avoiding Do Nothing) were not stronger when the effortful task was Add-3, compared to when it was Add-1 (see the Appendix for a figure of Wu and colleagues’ results).

#### Environmental context effects

There was a sharp increase in the proportion of harder task choices in the Lab compared to Online (Fig. 2). To quantify this effect, we used a logistic regression model predicting choice (hard or easy task) from experiment condition (Online or Lab). Across all comparisons, participants in the Lab experiment were 2.55 times more likely to choose the harder option than those in the Online experiment (*β*_lab_ = .938, *p* < 0.001, OR = 2.55; 95% CI: [2.30, 2.84]). The pattern of preferences between comparisons, however, remained the same for both Online and Lab experiments (see Fig. 2).

We also assessed how people’s propensity to choose the harder task changed across the course of the experiment. To assess whether there were differences between the Lab and Online experiments we conducted a generalised linear mixed model assessing choice predicted by trial number (1 – 60 across the experiment) and experiment (Lab or Online): *choice* ~ *trial* × *experiment* + (*trial* | *participant*). The results suggest that preferences for the harder task decreased across the experiment (*β*_trial_ = -.010, *p* < 0.019, OR = .990; 95% CI: [0.981 0.998]), but the rate of decrease across trials did not differ between the Lab and Online studies. See the Appendix for full analysis details.

### Self-report boredom and demand ratings

#### Difference between comparisons

**Fig. 3:**
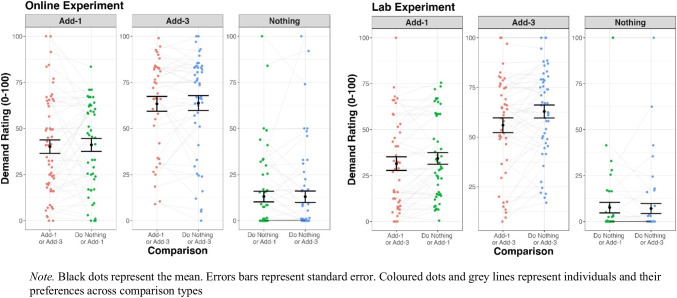
Self-report demand ratings for the online and lab experiments

**Fig. 4:**
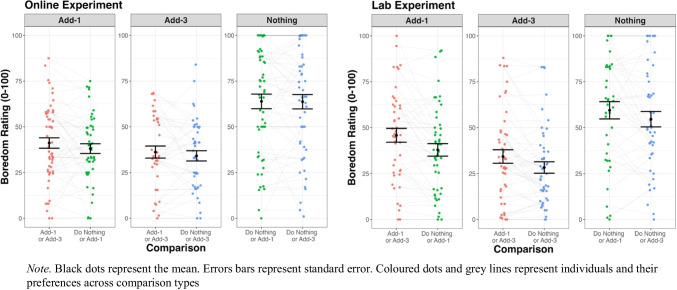
Self-report boredom ratings for the online and lab experiments

Self-report ratings for both the Online and Lab experiment are presented in Fig. 3 (demand ratings) and Fig. 4 (boredom ratings) below.

The primary interest was to assess whether participants’ ratings of a task type (e.g., Add-1) changed depending on the comparison (e.g., Do Nothing or Add-3). To assess the differences between these ratings we conducted repeated linear mixed model analyses assessing the effect of comparison on participants’ ratings for all tasks across both experiments. The nominal p-value was conservatively set at 0.008 (.05/6; 6 being the number of analyses conducted per experiment).

Online, there was no difference in demand ratings between comparisons for any of the task types (Add-1, Add-3, or Do-Nothing). In the Lab, however, both the Add-1 (*t*(41.14) = 2.80, *p* = .0077)^4^ and Add-3 (*t*(45.94) = 3.36, *p* = .0016) tasks were rated as being more demanding when the alternative was Do-Nothing as opposed to the other Add-N task.

There were no differences in boredom ratings between comparisons for the Online experiment. In the Lab, the Add-1 task was rated as *less* boring when the alternative was Do-Nothing as opposed to Add-3 (*t*(43.37) = -4.14, *p* = .0001); the effect of comparison on Add-3 task boredom ratings was analogous in direction, but this effect did not reach significance (*t*(46.35) = -2.73, *p* = .0089). See the Appendix for full details of all analyses.

It is also worth noting that the excluded participants tended to have higher boredom and demand ratings for both the Add-1 and Add-3 tasks. We report all demand and boredom ratings for included and excluded participants in the Appendix.

## Discussion

The current work investigated how people trade-off their competing aversions to cognitive effort and boredom. We also investigated the impact of environmental context by conducting the experiment both online, where myriad potential distractors exist, and in the lab, where conditions are highly regularised.

Regarding the effect of choice context, in alignment with previous work (Wu et al., 2023), our findings suggest people do not strive to merely minimise effort, but instead seek effort when the alternative imposes so little demand it evokes boredom. Consider people’s preference for the Add-3 task: in both of our experiments people’s preference for the Add-3 task was greater when the alternative was the effortless Do Nothing, as opposed to when it was the marginally demanding Add-1.

Choice context also had a small yet inconsistent effect on people’s self-reported experience of demand and boredom – small differences were only observed in the lab. These phenomenological differences, however, did not translate into on-task behavioural differences, with Add-1 and Add-3 accuracy remaining stable across comparisons for both the Online and Lab tasks. While participants’ choice preferences were affected by the context, it appears the amount of effort they exerted during their chosen task was unaffected by the alternative present.

It is also worth noting participants in our experiments generally made internally consistent (i.e., transitive) choices. While their aversion to effortful tasks, such as the Add-3, waned as the alternative became boring, we did not observe stronger ‘hard task’ preferences when the demanding task was Add-3 compared to the Add-1; this is in contrast to Wu et al. (2023) who observed effort seeking when the hard task was Add-3, but indifference when it was Add-1, despite preferring the Add-1 over the Add-3. This may be because participants in the current experiments experienced all possible alternatives (e.g., Nothing, Add-1, and Add-3) which led them to make internally consistent choices.

Regarding the effect of environmental context, we observed a large shift in preferences between the Online and Lab conditions. For any given trial, participants in the lab were 2.5 times more likely to choose the hard task than those who completed the study online. Task accuracy, however, was near equivalent in both experiments suggesting changes in task performance, which people are normally sensitive to (Embrey et al., 2023; Matthews et al., 2023; Westbrook et al., 2013), were not a moderating factor of these preferences. This suggests the change in the participants’ environment did not affect the effort they exerted during the Add-N tasks, nor merely introduce noise into their preferences, but rather systematically increased people’s aversion to the boring alternative and their subsequent willingness to choose effortful tasks.

Demand effects may have influenced people’s preferences, with the experimenter being physically present for parts of the Lab study (e.g., showing participants the testing rooms) compared to the Online study where no experimenter was present. The lack of demand avoidance observed in the Add-1 or Add-3 comparison in the Lab may be evidence that demand effects played a role; despite being told to choose whichever deck they preferred, participants in the lab may have chosen the harder option more frequently due to the presence of the experimenter.

Another relevant factor is that a participant has quite literally nothing to do during a Do-Nothing trial in the lab, whereas a Do-Nothing trial equates to “do anything else” in the online setting. While online participants don’t necessarily partake in other activities (we removed those who confessed), the opportunity to do so is undeniably greater. Given the theorised role of opportunity costs on people’s experiences of momentary effort and fatigue (Agrawal et al., 2022; Kurzban et al., 2013; Kurzban, 2016), it is perhaps unsurprising the presence of enjoyable alternatives altered choice preferences in our experiments.

On this point, it could be argued our results align with opportunity cost theories of effort given people’s preferences for mentally demanding tasks were dependent on the other opportunities present. We however caution against such a conclusion given the stronger predictions of opportunity cost theories (Kurzban et al., 2013) are not observed: people’s on-task behaviour (i.e., accuracy) is unaffected by the available alternative, despite it affecting their choice preferences. The novel prediction of opportunity cost theories, such as that proposed by Kurzban et al. (2013), is that the effort we expend towards a task’s goals is influenced by the value of the alternatives present. We however observe no such effect. The other opportunities available influence people’s willingness to *choose* effortful tasks in our experiments, but this isn’t a unique prediction of the opportunity cost theory – there is little profundity in the claim that what we choose depends on what’s offered.

On a methodological level, the current experiments inform the inferences researchers can draw from studies manipulating effort and boredom. In the present case, the difference in preferences between environmental contexts, if assessed in isolation, could lead to divergent conclusions. Consider participants’ choices in the Do-Nothing or Add-3 comparison: online, participants were averse to increased effort and preferred doing nothing; this contrasts with the lab, where participants preferred Add-3, avoiding boredom. While recent work (Uittenhove et al., 2023) argues it is more important *who* you test (an mTurk sample compared to a volunteer student sample) as opposed to *how* you test (online or in-person), the results of our experiments suggest this is not the case when effort and boredom aversion are the phenomena of interest.

## Limitations and future directions

There are some limitations to the current work that prevent us from drawing strong conclusions as to the causality of the observed effects. While participants did not have a choice between the Lab and Online studies (they were run sequentially in a short timeframe), participants were not randomly allocated and were aware of whether the study was online or in-person before enrolling. Participants who chose to partake in the Lab study may have differed in an important manner from those who enrolled in the Online study – for example, they may have been more ‘effort seeking’ or boredom averse. As we did not use any trait-based measures (e.g., Need for Cognition, see Cacioppo & Petty, 1982), we cannot ascertain how much of an effect this had on our results.

Another limitation is our inability to know what alternative tasks were present for participants in the Online study. While it was likely there were more distractions for those online (as there were none in the lab), we did not request details about where participants were situated nor what distractions were present. Future online studies would benefit from asking participants about their location, what alternatives were available and whether they engaged with them or not.

## Conclusion

In summary, regarding the cognitive costs that underlie effort avoidance, our results provide further evidence that our preferences for effortful tasks are not only dependent on the absolute computational costs and rewards of a task, but how these factors relate to the alternatives present. We, however, do not find evidence to support the notion that people’s willingness to expend effort towards a task depends on these alternatives (as predicted by Kurzban et al., 2013). 

More generally, the current experiments provide further support to Wu and colleagues’ (2023) finding that our aversion to effort is not absolute. We might generally be cognitive misers, but there is a limit to our parsimony.

## Supplementary information

Below is the link to the electronic supplementary material.

Supplementary file1 (DOCX 240 KB)

## Data Availability

All experimental data and analysis scripts can be accessed here: https://osf.io/e86fq/.

## Footnotes

1 We note that Wu et al. did conduct one of their studies in the lab (out of 13 total), but the use of different populations (Prolific vs. student sample), among other issues, makes it difficult to draw conclusions about the role of environmental context. We return to this point in the General discussion.

2 Due to a coding error, the Add-1 and Add-3 tasks lasted a total of 9 s, as opposed to 10 s. Participants were however informed that both tasks took the same amount of time (10 s) and no participants reported noticing a difference in times between the two tasks. This error was consistent across both Experiments and all participants.

3 One participant had a second phone on them, which they did not leave with the experimenter beforehand.

4 This result is no longer statistically significant when all participants are included – p-value of .0166.

## References

[CR1] Agrawal, M., Mattar, M. G., Cohen, J. D., & Daw, N. D. (2022). The temporal dynamics of opportunity costs: A normative account of cognitive fatigue and boredom. *Psychological Review,**129*(3), 564. 34383523 10.1037/rev0000309

[CR2] Ashburner, M., & Risko, E. F. (2022). On the influence of evaluation context on judgments of effort. *Journal of Experimental Psychology: Human Perception and Performance,**48*(8), 790–811. 35758975 10.1037/xhp0001026

[CR3] Bates, D., Mächler, M., Bolker, B., & Walker, S. (2015). Fitting linear mixed-effects models Usinglme4*. Journal of Statistical Software,* 67(1).

[CR4] Bench, S. W., & Lench, H. C. (2019). Boredom as a seeking state: Boredom prompts the pursuit of novel (even negative) experiences. *Emotion,**19*(2), 242. 29578745 10.1037/emo0000433

[CR5] Cacioppo, J. T., & Petty, R. E. (1982). The need for cognition. *Journal of personality and social psychology,**42*(1), 116.

[CR6] De Leeuw, J. R. (2015). jsPsych: A JavaScript library for creating behavioral experiments in a Web browser. *Behavior Research Methods,**47*, 1–12. 24683129 10.3758/s13428-014-0458-y

[CR7] Desjardins, S., Tang, R., Yip, S., Roy, M., & Otto, A. R. (2023). Context Effects in Cognitive Effort Evaluation. *PsyArXiv*. 10.3758/s13423-024-02547-8 39102161

[CR8] Eastwood, J. D., Frischen, A., Fenske, M. J., & Smilek, D. (2012). The unengaged mind defining boredom in terms of attention. *Perspectives on Psychological Science,**7*(5), 482–495. 26168505 10.1177/1745691612456044

[CR9] Elpidorou, A. (2018). The bored mind is a guiding mind: toward a regulatory theory of boredom. *Phenomenology and the Cognitive Sciences,**17*, 455–484.

[CR10] Embrey, J. R., Donkin, C., & Newell, B. R. (2023). Is all mental effort equal? The role of cognitive demand-type on effort avoidance. *Cognition,**236*, 105440. 36931050 10.1016/j.cognition.2023.105440

[CR11] Fiske, S. T., & Taylor, S. E. (1991). Social cognition. Mcgraw-Hill Book Company.

[CR12] Hull, C. L. (1943). The problem of intervening variables in molar behavior theory. *Psychological Review,**50*(3), 273.

[CR13] Ito, M., & Kawahara, J. I. (2017). Effect of the presence of a mobile phone during a spatial visual search. *Japanese Psychological Research,**59*(2), 188–198.

[CR14] Kahneman, D., Peavler, W. S., & Onuska, L. (1968). Effects of verbalization and incentive on the pupil response to mental activity. *Canadian Journal of Psychology,**22*(3), 186. 5658928 10.1037/h0082759

[CR15] Kool, W., McGuire, J. T., Rosen, Z. B., & Botvinick, M. M. (2010). Decision making and the avoidance of cognitive demand. *Journal of Experimental Psychology: General,**139*(4), 665. 20853993 10.1037/a0020198 PMC2970648

[CR16] Kurzban, R. (2016). The sense of effort. *Current Opinion in Psychology,**7*, 67–70.

[CR17] Kurzban, R., Duckworth, A., Kable, J. W., & Myers, J. (2013). An opportunity cost model of subjective effort and task performance. *Behavioral and Brain Sciences,**36*(6), 661–679. 24304775 10.1017/S0140525X12003196 PMC3856320

[CR18] Lorenc, E. S., Mallett, R., & Lewis-Peacock, J. A. (2021). Distraction in visual working memory: Resistance is not futile. *Trends in Cognitive Sciences,**25*(3), 228–239. 33397602 10.1016/j.tics.2020.12.004 PMC7878345

[CR19] Matthews, J., Pisauro, M. A., Jurgelis, M., Müller, T., Vassena, E., Chong, T. T. J., & Apps, M. A. (2023). Computational mechanisms underlying the dynamics of physical and cognitive fatigue. *Cognition,**240*, 105603. 37647742 10.1016/j.cognition.2023.105603

[CR20] Nederkoorn, C., Vancleef, L., Wilkenhöner, A., Claes, L., & Havermans, R. C. (2016). Self-inflicted pain out of boredom. *Psychiatry Research,**237*, 127–132. 26847946 10.1016/j.psychres.2016.01.063

[CR21] Oberauer, K., Lewandowsky, S., Awh, E., Brown, G. D., Conway, A., Cowan, N., ... & Ward, G. (2018). Benchmarks for models of short-term and working memory. *Psychological bulletin, 144(9)*, 885. 10.1037/bul0000153 30148379

[CR22] Oprea, R. (2020). What makes a rule complex? *American Economic Review,**110*(12), 3913–3951.

[CR23] Otto, A. R., Braem, S., Silvetti, M., & Vassena, E. (2022). Is the juice worth the squeeze? Learning the marginal value of mental effort over time. *Journal of Experimental Psychology: General.,**151*(10), 2324–2341. 35389742 10.1037/xge0001208

[CR24] Otto, A. R., & Vassena, E. (2021). It’s all relative: Reward-induced cognitive control modulation depends on context. *Journal of Experimental Psychology: General,**150*(2), 306. 32790463 10.1037/xge0000842

[CR25] Struk, A. A., Scholer, A. A., Danckert, J., & Seli, P. (2020). Rich environments, dull experiences: How environment can exacerbate the effect of constraint on the experience of boredom. *Cognition and Emotion,**34*(7), 1517–1523. 32401144 10.1080/02699931.2020.1763919

[CR26] Uittenhove, K., Jeanneret, S., & Vergauwe, E. (2023). From Lab-Testing to Web-Testing in Cognitive Research: Who You Test is More Important than how You Test. *Journal of Cognition,* 6(1). 10.5334/joc.259 PMC9854315 36721797

[CR27] Wagenmakers, E. J., & Farrell, S. (2004). AIC model selection using Akaike weights. *Psychonomic Bulletin & Review,**11*, 192–196. 15117008 10.3758/bf03206482

[CR28] Ward, A. F., Duke, K., Gneezy, A., & Bos, M. W. (2017). Brain drain: The mere presence of one’s own smartphone reduces available cognitive capacity. *Journal of the Association for Consumer Research,**2*(2), 140–154.

[CR29] Wu, R., Ferguson, A. M., & Inzlicht, M. (2023). Do humans prefer cognitive effort over doing nothing? *Journal of Experimental Psychology: General.,**152*(4), 1069–1079. 36355768 10.1037/xge0001320

