# Supplementary material for: Too hard, too easy, or just right? The effects of context on effort and boredom aversion
Source: Psychon Bull Rev. 2024 Jun 5;31(6):2801–10. doi: 10.3758/s13423-024-02528-x (PMC11680605; doi:10.3758/s13423-024-02528-x)
Supplement: Supplementary file 1 — Supplementary file1 (DOCX 240 KB) [file 13423_2024_2528_MOESM1_ESM.docx]

**Appendix**

This document contains the appendices for Embrey, Mason & Newell “Too Hard, Too Easy, Or Just Right? The Effects of Context on Effort and Boredom Aversion”. It contains the following sections:

1. Generalised linear mixed models for accuracy
2. Correlation matrices for individual choices preferences across comparison types
3. Comparisons to indifference (i.e., 50-50 choice) using repeated t-tests
4. Linear mixed models for self-report Boredom and Demand ratings
5. Effort choices across trials
6. Graphical figure of Wu et al. (2022) results
7. All analyses with full sample (no exclusions)
8. Boredom and demand ratings for excluded participants

**Section 1: Generalised linear mixed models for accuracy**

The baseline model contained only an intercept, *correct ~* 1 + (1 | *PID*), with the full model adding comparison as both a fixed and random effect, *correct ~ comparison* + (*comparison* | *PID* ). Models were assessed using Akaike weights.

Table S1 below shows the AIC and Akaike weights for all models for both experiments. The addition of comparison did not improve the fit of the model for either experiment for either of the two Add-N tasks.

**Table S1**

*Results for GLMM for accuracy across comparisons*

| Model | AIC | Akaike Weight |
| --- | --- | --- |
| Add-1 – Online Experiment | | |
| *correct ~* 1 + (1 \| *PID*) | 716.17 | .945 |
| *correct ~ comparison* + (comparison \| *PID* ) | 721.85 | .055 |
| Add-3 – Online Experiment | | |
| *correct ~* 1 + (1 \| *PID*) | 501.61 | .905 |
| *correct ~ comparison* + (comparison \| *PID* ) | 506.19 | .095 |
| Add-1 – Lab Experiment | | |
| *correct ~* 1 + (1 \| *PID*) | 642.07 | .687 |
| *correct ~ comparison* + (1 \| *PID* )* | 643.64 | .313 |
| Add-3 – Lab Experiment | | |
| *correct ~* 1 + (1 \| *PID*) | 965.20 | .910 |
| *correct ~ comparison* + (comparison \| *PID* ) | 969.83 | .090 |

*Note. **The ‘full’ model for the Add-1 in the Lab experiment does not contain *comparison* as a random slope. This is because the model containing a random slope did not converge and hence the model was simplified.

**Section 2: Correlation matrices for choices preferences across comparison types**

The below tables are Pearson correlation matrices which show the relationship between hard choice preferences across comparison types. For example, how well does someone’s hard choice preferences in Add-1 or Add-3 predict their hard choice preferences in the Do Nothing or Add-1 comparison.

**Table S2a**

*Pearson correlations for hard choice preferences between comparison types for the Online Experiment*

|  | Add-1 or Add-3 | Nothing or Add-1 |
| --- | --- | --- |
| Nothing or Add-1 | 0.62 |  |
| Nothing or Add-3 | 0.72 | 0.82 |

**Table S2b**

|  | Add-1 or Add-3 | Nothing or Add-1 |
| --- | --- | --- |
| Nothing or Add-1 | 0.39 |  |
| Nothing or Add-3 | 0.77 | 0.55 |

*Pearson correlations for hard choice preferences between comparison types for the Lab Experiment*

**Section 3: Comparisons to indifference using repeated t-tests**

Following Wu et al. (2022) we also compared preferences for each comparison to indifference via repeated t-tests. The following reported p-values are uncorrected for multiple comparisons. For Do-Nothing or Add-1, participants were indifferent Online (*M* = .485, *t*(48) = -.360, *p* = .720) and preferred Add-1 in the Lab (*M* = .715, *t*(50) = 5.682, *p* < .001); for Do Nothing or Add-3 participants were effort averse Online (*M* = .376, *t*(48) = -2.915, *p* = .005) and indifferent in the Lab (*M* = .566, *t*(50) = 1.968, *p* = .055); for the Add-1 or Add-3 participants Online were effort averse (*M* = .268, *t*(48) = -6.190, *p* < .001) and indifferent in the Lab (*M* = .529, *t*(50) = .686, *p* = .496).

**Section 4: Repeated linear mixed models for self-report ratings**

As indicated in the main text, we ran multiple linear mixed models assessing whether people’s self-report ratings (both boredom and demand) of the tasks differed by comparison. We set the nominal p-value at .008 given we were conducting 6 analyses per experiment.

All models were of the form *rating* ~ *comparison* + (1 | *PID* ) where the rating was either a boredom or demand rating. We did not fit random slopes for comparison give the relative dearth of data (max 2 data points per subject).

Below, we report the results per experiment (lab or online) and for each rating type (boredom or demand) in table format. The degrees of freedom are lower in some groups due to some participants never attempting one of the two tasks in a comparison and therefore no self-report rating exists for that participant and task type.

**Table S4a**

*Mixed model outputs for demand ratings for both Online and Lab experiments*

| Task Type | df | t value | p-value |
| --- | --- | --- | --- |
| Online Experiment | | | |
| Add-1 | 48.715 | .253 | .801 |
| Add-3 | 38.146 | -.162 | .872 |
| Do-Nothing | 54.205 | -.215 | .831 |
| Lab Experiment | | | |
| Add-1 | 42.143 | 2.801 | .0077 |
| Add-3 | 45.938 | 3.355 | .0016 |
| Do-Nothing | 39.657 | -.208 | .836 |

**Table S4b**

*Mixed model outputs for boredom ratings for both Online and Lab experiments*

| Task Type | df | t value | p-value |
| --- | --- | --- | --- |
| Online Experiment | | | |
| Add-1 | 48.658 | -1.28 | .207 |
| Add-3 | 38.866 | -.841 | .405 |
| Do-Nothing | 54.237 | -.217 | .829 |
| Lab Experiment | | | |
| Add-1 | 43.370 | -4.139 | .0002 |
| Add-3 | 46.353 | -2.733 | .0089 |
| Do-Nothing | 38.136 | -2.137 | .0390 |

**Section 5: Effort choices across trials**

To assess whether there were differences between the Lab and Online experiments we conducted a generalised linear mixed model assessing choice (easier or harder option, collapsing across comparison type) as predicted by trial number (1 – 60 across the experiment) and experiment (Lab or Online), choice ~ trial*experiment + (trial | participant). We started with a baseline model containing only an intercept, and compared this to a model containing *trial* as a fixed factor (and a random slope of *trial*), to a full model containing *trial* and *experiment* and their interaction.

Model comparison (lowest AIC) found the full model to be the best fitting and we report the summary of the parameter estimates in Table S5 below. It is however also worth noting that the model containing *trial* only was a significant improvement on the baseline model, indicating that *trial* affected participant’s propensity to choose the effortful task, with their tendency to choose the effortful task declining over time. The rate of decrease in harder task choices however is not significantly different between the Online and Lab Experiment.

**Table S5**

*Summary of generalised mixed models to assess choices across trials*

| Parameter | Estimate | z value | p-value |
| --- | --- | --- | --- |
| *choice ~ trial + (trial \| participant)*: AIC = 6489.4 | | | |
| Intercept | 0.189 |  |  |
| Trial | -0.010 | -2.356 | 0.0185* |
| *choice ~ trial*experiment + (trial \| participant)*: AIC = 6476.4 | | | |
| Intercept | 0.742 |  |  |
| Trial | -0.004 | 0.006 | 0.498 |
| Experiment (Online) | 1.121 | 0.328 | <0.001* |
| Interaction | -0.013 | 0.009 | 0.147 |

*Note.* Estimates are on the log-odds scale.

**Figure S5**

*Choices across trials for the Online and Lab experiments*

**
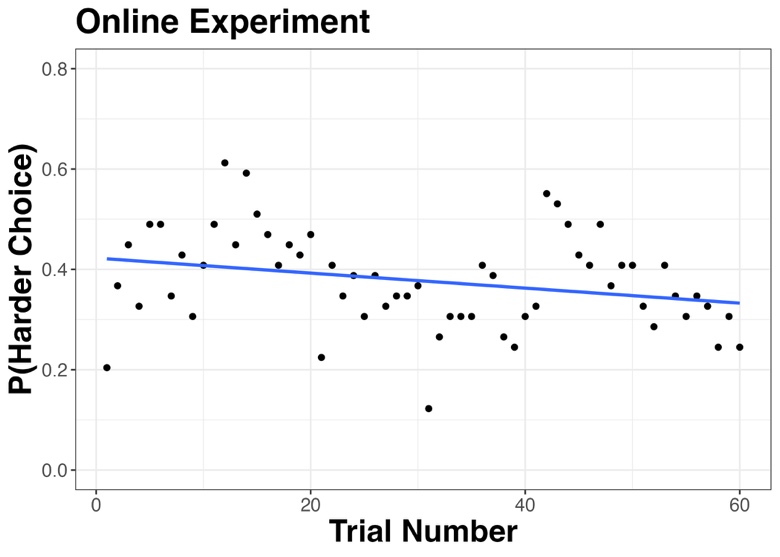

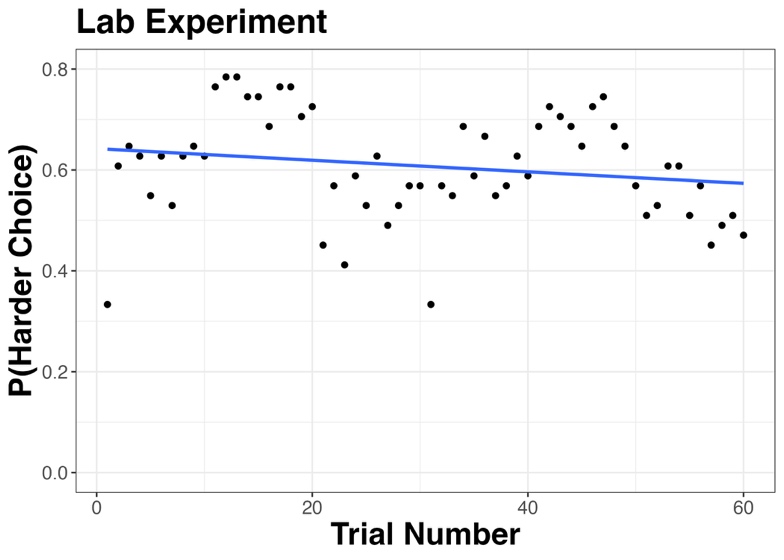
**

**Section 6: Graphical figure of Wu et al. (2022) results**

Below, in Figure S5, we report the summarised results of Wu et al. (2022) using their reported means and standard errors to depict the results from the relevant experiments (Pilot – Experiment 5). The starkest difference with the work we present is the preferences for the Do Nothing or Add-3 comparison relative to Do Nothing or Add-1. In our experiments, the preference for the harder task is significantly less when the hard task is Add-3 compared to Add-1. Wu et al. (2022), however, observe the opposite pattern of results. This is presumably due to Wu et al. (2022) using separate individuals for each experiment (comparison), whereas participants in our experiments completed all three possible comparisons.

**Figure S5**

*Average choice preferences for participants in Wu et al. (2022)*


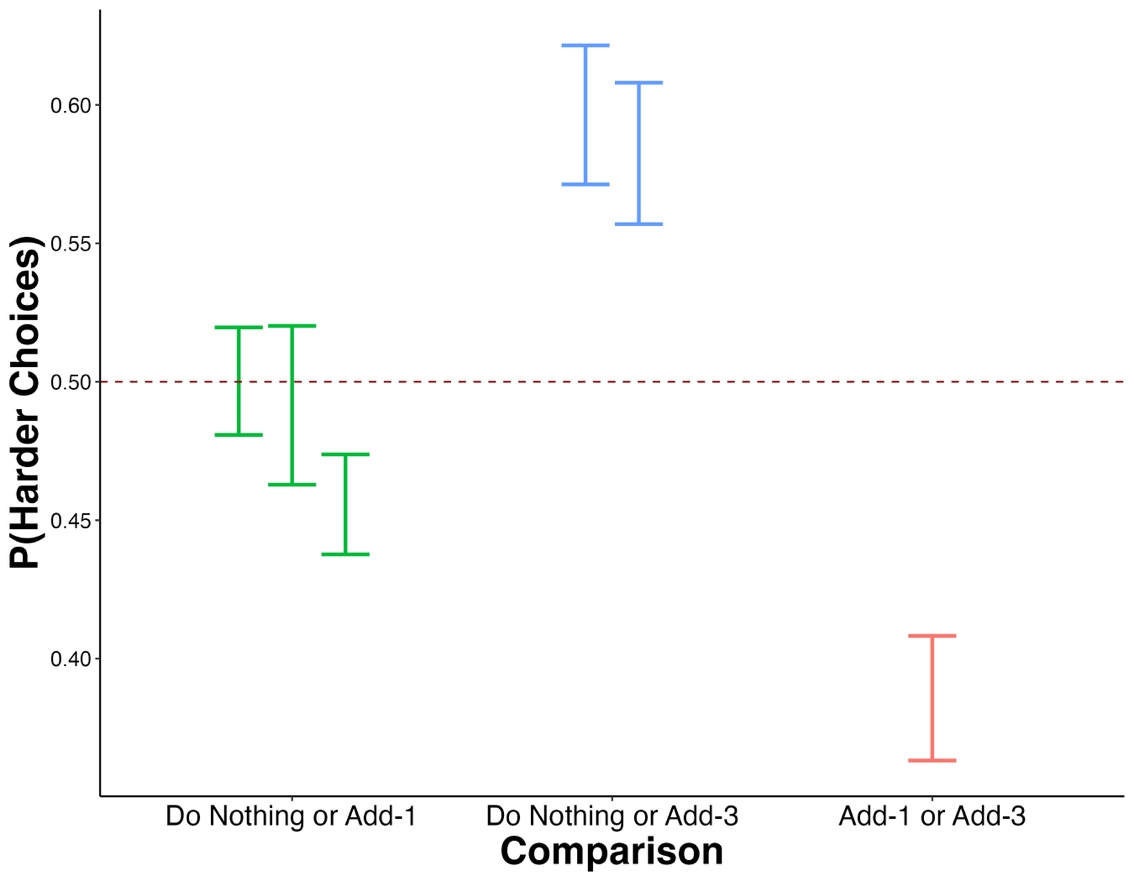


*Note.* Each separate set of error bars represents an individual experiment with the colour indicating what the two offered tasks were for that experiment. The error bars are ±1 SEM from the mean. The dashed line represents the indifference point.

**Section 7: All analyses with full sample (no exclusions)**

***Accuracy analysis***

The below results are the same as those reported in Section 1 of the Appendix but no exclusion criteria have been used (i.e., the full sample is used). This results in one very minor change: the difference between Add-1 demand rating in the Lab is no longer significant (p value shifts to .0166, from .0077, where the nominal p-value was .008).

**Table S7a**

*Results for GLMM for accuracy across comparisons (full sample)*

| Model | AIC | Akaike Weight |
| --- | --- | --- |
| Add-1 – Online Experiment | | |
| *correct ~* 1 + (1 \| *PID*) | 1107.31 | .934 |
| *correct ~ comparison* + (comparison \| *PID* ) | 1112.61 | .066 |
| Add-3 – Online Experiment | | |
| *correct ~* 1 + (1 \| *PID*) | 604.30 | .930 |
| *correct ~ comparison* + (comparison \| *PID* ) | 609.48 | .070 |
| Add-1 – Lab Experiment | | |
| *correct ~* 1 + (1 \| *PID*) | 759.67 | .444 |
| *correct ~ comparison* + (1 \| *PID* )* | 759.17 | .556 |
| Add-3 – Lab Experiment | | |
| *correct ~* 1 + (1 \| *PID*) | 1022.84 | .923 |
| *correct ~ comparison* + (comparison \| *PID* ) | 1027.81 | .077 |

*Note. **The ‘full’ model for the Add-1 in the Lab experiment does not contain *comparison* as a random slope. This is because the model containing a random slope did not converge and hence the model was simplified.

***Demand selection task preferences***

**Choice context effects.**

The below is the same analysis as reported in the Results section for preferences in the demand selection task. Here, however, the full sample is retained. There are no meaningful differences in the results or our interpretations.

**Table S7b**

*Results for GLMM: Online and Lab Experiments (full sample)*

| Model | AIC | Akaike Weight |
| --- | --- | --- |
| Online Experiment | | |
| *choice ~* 1 *+* (1*\| participant*) | 4126.00 | <.001 |
| *choice ~ comparison +* (1*\| participant*) | 3955.85 | >.999 |
| Lab Experiment | | |
| *choice ~* 1 *+* (1*\| participant*) | 3789.97 | <.001 |
| *choice ~ comparison +* (1*\| participant*) | 3647.55 | >.999 |

**Table S7c**

*Results for pairwise contrasts: Online and Lab Experiments (full sample)*

| Contrast | Estimate (SE) | z-score | *p*-value |
| --- | --- | --- | --- |
| Online Experiment | | | |
| (A-1 or A-3) – (Nothing or A-1) | -1.308 (.102) | -12.839 | <.001 |
| (A-1 or A-3) – (Nothing or A-3) | -.614 (.102) | -6.046 | <.001 |
| (Nothing or A-1) – (Nothing or A-3) | .694 (.096) | 7.204 | <.001 |
| Lab Experiment | | | |
| (A-1 or A-3) – (Nothing or A-1) | -1.171 (.105) | -11.191 | <.001 |
| (A-1 or A-3) – (Nothing or A-3) | -.214 (.098) | -2.178 | 0.088 |
| (Nothing or A-1) – (Nothing or A-3) | .957 (.104) | 9.180 | <.001 |

Note. Estimates are given on the log odds ratio, not the response variable scale. p-values are Bonferroni corrected.

**Environmental context effect.**

The analysis method is the same as that reported in the paper except the full sample is retained here.

Across all comparisons, participants in the Lab experiment were 2.95 times more likely to choose the harder option than those in the Online experiment (*β*_lab_ = 1.082, *p* < 0.001, OR = 2.95; 95% CI: [2.69, 3.24]).

***Self-report boredom and demand ratings***

The below is the analyses for the self-report data without removals. The only notable change is that the analysis of comparison type for Add-1 demand ratings is no longer significantly different in the lab: the p-value is now .0166, as opposed to .007 when the exclusion criteria is applied. The nominal p-value after Bonferroni correction is .008.

**Table S7d**

*Mixed model outputs for demand ratings for both Online and Lab experiments (full sample)*

| Task Type | df | t value | p-value |
| --- | --- | --- | --- |
| Online Experiment | | | |
| Add-1 | 58.277 | .638 | .526 |
| Add-3 | 44.340 | -.592 | .557 |
| Do-Nothing | 67.241 | -.535 | .594 |
| Lab Experiment | | | |
| Add-1 | 47.486 | 2.483 | .0166 |
| Add-3 | 47.933 | 3.332 | .002 |
| Do-Nothing | 44.656 | .305 | .7620 |

**Table S7e**

*Mixed model outputs for boredom ratings for both Online and Lab experiments (full sample)*

| Task Type | df | t value | p-value |
| --- | --- | --- | --- |
| Online Experiment | | | |
| Add-1 | 59.133 | -1.278 | .206 |
| Add-3 | 44.660 | -.833 | .410 |
| Do-Nothing | 67.249 | .597 | .553 |
| Lab Experiment | | | |
| Add-1 | 48.779 | -3.418 | .001 |
| Add-3 | 48.720 | -3.058 | .004 |
| Do-Nothing | 42.423 | -1.523 | .135 |

**Section 8: Boredom and demand ratings for excluded participants**

Here we report the average demand and boredom ratings for participants who were excluded in both the Online and Lab experiments. On average, the boredom and demand ratings for the two Add-N tasks (Add-1 and Add-3) tend to be higher for the removed participants (either due to low accuracy or due to completing other tasks) than the participants included in the main analysis. Average ratings for included and excluded participants can be seen below in Tables S8a and S8b.

It is hard to determine the causal direction of the relationship between people’s reported phenomenology during the tasks and their behaviour (i.e., accuracy and being distracted), but given the typically observed relationship between willingness to perform effortful tasks and performance (Embrey et al., 2023; Mathews et al., 2023; Westbrook et al., 2013) we assume that poor task performance likely led to increased disengagement and increased demand and boredom ratings.

**Table S8a**

*Boredom and demand ratings for the Online study for included and excluded participants*

| Rating Type | Task Type | Included –  Rating (SD) | Excluded –  Rating (SD) |
| --- | --- | --- | --- |
| Boredom | Add-1 | 38.5 (20.5) | 46.1 (15.1) |
| Boredom | Add-3 | 34.0 (20.1) | 41.5 (20.6) |
| Boredom | Do-Nothing | 63.8 (29.8) | 61.0 (30.9) |
| Demand | Add-1 | 38.4 (25.7) | 51.8 (24.9) |
| Demand | Add-3 | 62.3 (27.1) | 75.8 (16.5) |
| Demand | Do-Nothing | 12.1 (20.6) | 16.4 (26.5) |

**Table S8a**

*Boredom and demand ratings for the Lab study for included and excluded participants*

| Rating Type | Task Type | Included –  Rating (SD) | Excluded –  Rating (SD) |
| --- | --- | --- | --- |
| Boredom | Add-1 | 41.7 (24.7) | 40.2 (21.9) |
| Boredom | Add-3 | 31.3 (23.6) | 60.6 (23.2) |
| Boredom | Do-Nothing | 56.8 (29.2) | 51.6 (40.6) |
| Demand | Add-1 | 33.0 (23.5) | 60.7 (24.9) |
| Demand | Add-3 | 59.4 (23.9) | 77.3 (18.8) |
| Demand | Do-Nothing | 7.3 (18.4) | 11.3 (17.5) |
